# Visual Feedback Modulates Aftereffects and Electrophysiological Markers of Prism Adaptation

**DOI:** 10.3389/fnhum.2020.00138

**Published:** 2020-04-17

**Authors:** Jasmine R. Aziz, Stephane J. MacLean, Olave E. Krigolson, Gail A. Eskes

**Affiliations:** ^1^Cognitive Health and Recovery Research Lab, Departments of Psychiatry, Psychology and Neuroscience, Brain Repair Centre, Dalhousie University, Halifax, NS, Canada; ^2^Centre for Biomedical Research, University of Victoria, Victoria, BC, Canada

**Keywords:** prism adaptation (PA), visuo-spatial neglect, strategic recalibration, spatial realignment, visual feedback, event-related potentials (ERP), P300, feedback feedback-related negativity (FRN)

## Abstract

Prism adaptation (PA) is both a model for visuomotor learning and a promising treatment for visuospatial neglect after stroke. The task involves reaching for targets while prism glasses horizontally displace the visual field. Adaptation is hypothesized to occur through two processes: strategic recalibration, a rapid self-correction of pointing errors; and spatial realignment, a more gradual adjustment of visuomotor reference frames that produce prism aftereffects (i.e., reaching errors upon glasses removal in the direction opposite to the visual shift). While aftereffects can ameliorate neglect, not all patients respond to PA, and the neural mechanisms underlying successful adaptation are unclear. We investigated the feedback-related negativity (FRN) and the P300 event-related potential (ERP) components as candidate markers of strategic recalibration and spatial realignment, respectively. Healthy young adults wore prism glasses and performed memory-guided reaching toward vertical-line targets. ERPs were recorded in response to three different between-subject error feedback conditions at screen-touch: view of hand and target (Experiment 1), view of hand only (Experiment 2), or view of lines to mark target and hand position (view of hand occluded; Experiment 3). Conditions involving a direct view of the hand-produced stronger aftereffects than indirect hand feedback, and also evoked a P300 that decreased in amplitude as adaptation proceeded. Conversely, the FRN was only seen in conditions involving target feedback, even when aftereffects were smaller. Since conditions producing stronger aftereffects were associated with a phase-sensitive P300, this component may index a “context-updating” realignment process critical for strong aftereffects, whereas the FRN may reflect an error monitoring process related to strategic recalibration.

## Introduction

Prism adaptation (PA) is a visuomotor task that demonstrates the brain’s adaptation to changes in visually perceived coordinates of objects in space (Redding et al., 2005). During PA, participants perform goal-directed reaching towards targets while wearing glasses that displace their visual field laterally. Initially, participants make large pointing errors in the direction of the prismatic shift (i.e., direct effects), after which they gradually adapt to the shift and become more accurate. Upon glasses removal, they make errors in the direction opposite to the preceding prismatic displacement (i.e., aftereffects), providing a measure of the strength of adaptation (Redding et al., 2005).

While PA is often used as a model of visuomotor learning (e.g., Henriques and Cressman, 2012), it is also a promising treatment for visuospatial neglect: an attentional disorder that affects approximately half of individuals with right-hemisphere stroke and involves difficulty attending, orienting, and responding to stimuli on the left side of the patient’s space and/or body (Rossetti et al., 1998; Buxbaum et al., 2004). Specifically, the leftward aftereffects following right-shifting PA can improve performance on visual scanning and reaching tasks (e.g., Rossetti et al., 1998; Nys et al., 2008), as well as in daily activities such as postural balancing or wheelchair driving (Tilikete et al., 2001; Jacquin-Courtois et al., 2008). While promising, PA’s therapeutic effects are inconsistent across patients (e.g., Nys et al., 2008; Turton et al., 2010). Acquiring a better understanding of the neural processes that give rise to PA aftereffects, and presumably contribute to improvements in neglect symptoms, might help optimize the therapy for clinical use.

Redding and Wallace (1996) theory of PA describes two separate adaptation mechanisms: “strategic recalibration” and “spatial realignment.” Under this model, recalibration reflects the rapid self-correction of pointing errors by adjusting the motor plan within a shifted work-space. Realignment, on the other hand, reflects a slower adjustment of spatial reference frames to reconcile the visuomotor discrepancy. Some studies suggest that realignment is more critical in producing PA aftereffects than recalibration. For example, participants who were exposed to prisms in multi-step increments such that the shift was not noticeable (and thus may not have been engaging in strategic error correction) experienced larger aftereffects than participants who were exposed to the same degree of shift in a single-step increment (Michel et al., 2007). Further, Redding and Wallace (1993) probed the magnitude of aftereffects across 10 trial increments during a PA block consisting of 60 trials and showed that aftereffects increased in later trials despite participants no longer engaging in any substantial error correction. Taken together, these results suggest that recalibration may not be as critical to producing aftereffects; furthermore, finding ways to identify and enhance realignment processes could have important implications for theories of PA and its therapeutic efficacy.

To better understand the contributions of recalibration and realignment to aftereffects, two studies have used electroencephalography (EEG) to investigate event-related potential (ERP) components evoked by the onset of error feedback during prism exposure. Vocat et al. (2011) identified an error-related negativity (ERN) and an error positivity (Pe) during PA that increased in amplitude concomitantly with the size of reaching errors. MacLean et al. (2015) corroborated the findings of Vocat et al. (2011) by also detecting an ERN sensitive to error size during PA. The ERN typically peaks at 50–100 ms post-response onset over fronto-central scalp electrodes, and it is thought to reflect the earliest internal evaluation that a response is worse than predicted (Holroyd and Coles, 2002). The Pe, by contrast, typically peaks at 100–200 ms post-response onset over central scalp electrodes and reflects subsequent evaluation of the error (Falkenstein et al., 1991; Wessel, 2012). Taken together, these studies suggest that pointing errors during prism exposure evoke neural processes of error evaluation, as indexed by the ERN and Pe (Krigolson et al., 2008; Vocat et al., 2011; MacLean et al., 2015).

While Vocat et al. (2011) examined error correction over 12 reaching trials of PA exposure, MacLean et al. (2015) increased the exposure length to 45 trials to facilitate investigation of realignment processes (Redding and Wallace, 1993). MacLean et al. (2015) identified a P300 component that decreased in amplitude throughout adaptation and was independent of pointing accuracy. The P300 is a centro-parietal component peaking at 300 ms that has been broadly linked to the attentional processing of task-relevant stimuli (Polich and Kok, 1995). Relevant to PA, the context-updating hypothesis of the P300 proposes that the P300 reflects an updating of participants’ inner working model of the environment (Donchin and Coles, 1988, 1998). According to this model, reductions in P300 amplitude across prism exposure could reflect the successful adjustment of internal spatial coordinates to accommodate the shift in the visual environment induced by the prisms. Overall, Vocat et al. (2011) and MacLean et al. (2015) showed that feedback during PA can elicit brain potentials sensitive to accuracy (e.g., Pe, ERN) and phase of adaptation (i.e., the P300).

An early hypothesis proposed by MacLean et al. (2015) was that the above-noted accuracy-sensitive components may reflect a neural process contributing to recalibration, whereas the phase-sensitive P300 may reflect a neural process contributing to realignment and may underly aftereffects. This hypothesis, however, requires further investigation. Our approach was to create PA feedback conditions that would favor either recalibration or realignment and determine whether the ERPs presumed to underly these processes were evoked.

Our justifications for the feedback conditions used in the present study are as follows. First, recalibration involves self-correction of the reaching trajectory that relies on receiving information about pointing accuracy (Redding and Wallace, 1996). Thus, we used memory-guided reaching and created feedback conditions with explicit error information at screen touch (i.e., participants saw a re-appearance of the target line to show a discrepancy with the hand position), or no explicit error information (i.e., participants only saw their hand position with no reappearance of the target line), expecting that the former would facilitate recalibration and evoke accuracy-sensitive ERPs. Although prior studies measured the ERN (Vocat et al., 2011; MacLean et al., 2015), our hypotheses focused on the feedback-related negativity (FRN), a fronto-central component peaking approximately 200–300 ms post-onset of error feedback (Miltner et al., 1997; Holroyd and Coles, 2002). The FRN was chosen because it is more closely associated with responses time-locked to error feedback from the *external* environment, which is what was manipulated in the present study; by contrast, the ERN has been described as a measure of error evaluation based on the *internal* environment (e.g., efference copy of the motor command; see Miltner et al., 1997; Holroyd and Coles, 2002; Holroyd et al., 2004). Further, the FRN has also been shown to be sensitive to the magnitude of endpoint errors during reaching tasks (Krigolson et al., 2008; Anguera et al., 2009).

To create feedback conditions that may modulate realignment, we drew on past studies suggesting that PA aftereffects are stronger when participants have a direct view of their hand at the termination of their reach than when they view a “symbolic” hand (e.g., cursor icon; Clower and Boussaoud, 2000; Wilms and Malá, 2010; Veilleux and Proteau, 2015). Explanations of this phenomenon referenced Welch and Warren (1980) “object unity assumption,” which states that in situations of misalignment between visual and proprioceptive inputs, participants can better adapt to the mismatch if they perceive both inputs as originating from the same source (i.e., their own body). In terms of PA, participants may be experiencing stronger aftereffects when they receive direct hand feedback because this allows them to better resolve the discrepancy between their proprioceptive “felt” hand position and visually perceived hand position, facilitating spatial realignment (Redding and Wallace, 2006). Thus, we created feedback conditions with a direct view of the hand (i.e., terminal exposure), or indirect view of the hand (i.e., participant’s hand was blocked by an occlusion board and they instead saw a vertical line referring to their hand position below the board), expecting that conditions with a direct view of the hand would facilitate realignment (as measured by larger aftereffects, Redding et al., 2005) and evoke a phase-sensitive P300.

In summary, the purpose of the present study was to investigate the neural processes contributing to recalibration and realignment during PA. Healthy young participants performed memory-guided reaching for vertical-line targets on a touch screen for alternating 60-trial blocks of prism and sham (i.e., clear) exposure. During PA, participants wore an EEG cap that was used to measure ERPs time-locked to visual feedback at screen touch. Aftereffects were measured using proprioceptive visual straight-ahead pointing (PVSA; Redding et al., 2005). We report results from three sequential experiments differing in the type of error feedback provided at the screen touch. In Experiment 1, participants saw both their hand and a re-appearance of the target line, in the replication of MacLean et al. (2015). Here, it was hypothesized that feedback would evoke an accuracy-sensitive FRN, a phase-sensitive P300, and strong aftereffects. Next, Experiments 2 and 3 sought to dismantle the two feedback components. In Experiment 2, participants saw their hand only, with no reappearance of the target line. Here, it was hypothesized that feedback would not evoke an FRN, but would still evoke a phase-sensitive P300 and produce strong aftereffects. In Experiment 3, participants could not directly view their hand (i.e., the hand was fully occluded) and instead saw re-appearance of the black target line and a second lighter gray vertical line that referred to the hand position below the board. Here, it was hypothesized that feedback would evoke an FRN, but no phase-sensitive P300 and weaker aftereffects than the other two experiments. Obtaining this pattern of results would support the theory that the neural process underlying the P300 contributes to a realignment process important for strong aftereffects, whereas the neural process underlying the FRN contributes to a recalibration process that is dependent on error feedback, but less essential to producing strong aftereffects.

## Materials and Methods

### Participants

The study recruited 67 young adult participants divided across three experiments (see “Design and Procedure” section). Table 1 displays sample size, age, gender, and handedness of participants by experiment after exclusions (see “Results” section for more details). All participants were students at Dalhousie University who provided informed consent consistent with requirements from the Nova Scotia Health Authority Research Ethics Board. All participants reported normal or corrected-to-normal vision, no neurological illness, no current use of medications affecting cognitive performance, and no upper-limb impairment preventing reaching movements with their dominant arm.

**Table 1:** Participant demographics by experiment after exclusions.

Experiment	*N*	Age		Gender (female/male)	Handedness (left/right)
		*M*	*SD*		
1: View of target and hand	18	19.50	1.70	16/2	3/15
2: View of hand only					
Behavioral data	21	19.95	1.91	20/1	1/20
EEG data	22	20.00	1.90	21/1	2/20
3: View of target and indirect hand					
Behavioral data	21	19.30	1.30	17/4	1/20
EEG data	22	19.70	1.40	18/4	1/21

*Note. Experiment 1 had two exclusions, Experiment 2 had one exclusion for behavioral data, and Experiment 3 had four exclusions for behavioral data and three exclusions for EEG data (see "Results" section for more details)*.

### Apparatus

Across experiments, participants were seated at an adjustable chair in front of a 28” touchscreen monitor (Intellitouch, USA). A chinrest was locked to the edge of the table to maintain participants at a distance of 48 cm from the screen, and at a height that kept their gaze in line with the center of the monitor. A keyboard (used to record response onset) was placed 10 cm in front of the chinrest with the spacebar in line with the center of the monitor. Speakers (used to emit a go-cue) were placed on the left and right of the monitor. A black horizontal occlusion board extending from the chinrest prevented participants from viewing their reaching movement until the last 3 cm immediately before screen touch, a set-up known as terminal exposure (Facchin et al., 2018). For certain conditions, the occlusion board was extended to prevent any vision of the limb to measure aftereffects (i.e., proprioceptive visual straight-ahead pointing, PVSA; Redding et al., 2005) or to prevent view of hand feedback (Experiment 3).

The PA experiment was designed in MATLAB (MathWorks, 2014) using the Psychophysics Toolbox extension (Brainard, 1997). Reaching targets consisted of vertical black lines that spanned the entire height of the monitor and were approximately 1.3 cm wide. On each PA trial, the target appeared randomly in one of four possible locations along the screen’s horizontal axis: (1) 5 cm to the left of the screen’s center; (2) 2.5 cm left of the screen’s center; (3) 2.5 cm right of the screen’s center; and (4) 5 cm right of the screen’s center. On PVSA trials, the target was centrally located. Depending on the block, participants wore a set of glasses with either clear lenses or Fresnel prism lenses (Insight Optometry Group, Halifax NS). Both left- and right-shifting prism glasses were used to prevent reaching strategies due to expectation of shift direction, and both induced a 30 diopter (or 17.7°) visual shift.

As participants completed the PA task, EEG data were collected from 64 electrodes in a standard 10-20 layout, using Brain Vision PyCorder (Brain Products, Germany). The EEG was recorded with an average reference, sampled at 500 Hz, amplified (ActiCHamp, Brain Products, Germany), and filtered online through an 8 kHz anti-aliasing filter. The impedance at each electrode was kept below 20 kΩ.

### Design and Procedure

**Figure 1:**
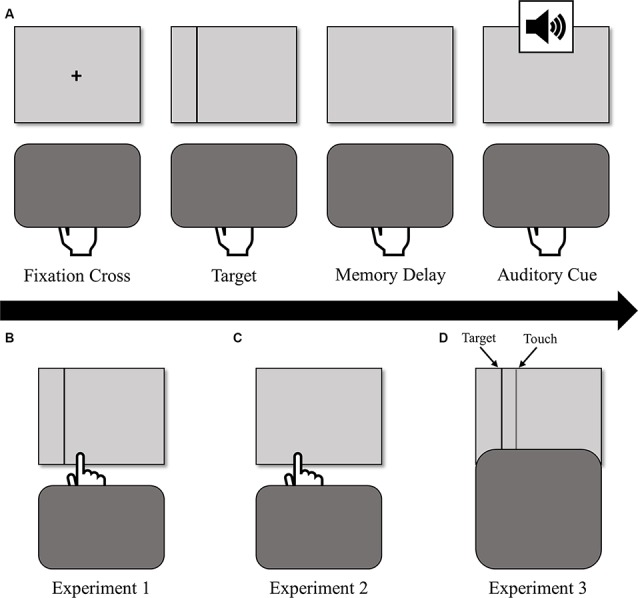
**(A)** The sequence of events for a single prism adaptation (PA) trial (top row). Participants held down the space bar, during which they viewed a fixation cross, the target, and a memory delay, after which an auditory cue prompted them to reach. Participants received different types of visual feedback at screen touch depending on the experiment: **(B)** in Experiment 1, participants saw their hand and a re-appearance of the target line (bottom-left); **(C)** in Experiment 2, participants saw their hand only (bottom-center); **(D)** in Experiment 3, participants could not directly view their hand (i.e., full occlusion) and instead saw re-appearance of the black target line and a second gray vertical line that referred to the hand position below the board (bottom-right).

This study consisted of three experiments differing in the type of visual feedback provided at screen touch during PA blocks: in Experiment 1 (*N* = 20), participants saw their hand and a re-appearance of the target line with terminal exposure; in Experiment 2 (*N* = 22), participants saw their hand only with the same terminal exposure; and in Experiment 3 (*N* = 25), participants could not directly view their hand (i.e., full occlusion) and instead saw re-appearance of the black target line and a second light gray vertical line that referred to the hand position below the board (see Figure 1 for a visual representation of the three feedback conditions).

Every experiment had the same 14-block structure. Participants began with a 10-trial PVSA block and 60-trial baseline sham block (both with clear lenses), which provided a measure of participants’ reaching accuracy excluding any PA aftereffects. Participants then completed a series of PA blocks with either left- or right-shifting prisms. There were four 60-trial prism exposure blocks in total per experiment, two with left-shifting prisms and two with right-shifting prisms. In Experiments 1 and 3, two prism blocks had immediate visual feedback (i.e., at screen touch), and two blocks had delayed feedback (i.e., 750–850 ms after screen touch). These four blocks were presented in a randomized order for each participant. Only results from the blocks with immediate feedback are presented in this article. The prism blocks for Experiment 2 had a similar structure in terms of left- and right-shifting PA blocks, but two blocks had “hand only” feedback (used in the present study) and two blocks had full target feedback (i.e., the target remained visible throughout the entire reach, as in MacLean et al., 2015). Given that the full target feedback condition yielded similar results to past work (MacLean et al., 2015), and did not involve memory-guided reaching, the present study focused on results from the “hand only” feedback condition of Experiment 2. After each PA block, participants completed a PVSA block that measured the strength of aftereffects produced by the adaptation, and a 60-trial sham block to de-adapt from the prism exposure. Overall, this block design yielded one 60-trial PA block, one 60-trial de-adaptation (sham) block, and one 10-trial post-prism PVSA block for each shift direction (left or right) per participant, in addition to the baseline PVSA and sham blocks.

Participants initiated each PA reaching trial by pressing down the spacebar with the index finger of their dominant hand. After holding down the spacebar for 500 ms, a fixation cross appeared for 400–600 ms at the center of the screen and was immediately followed by a vertical line target, which remained on the screen for 700–900 ms. After target disappearance, the screen remained blank for 1,000–1,200 ms before the participants heard an auditory cue (1,000 Hz, 0.05 s, 30 dB) that prompted them to release the spacebar and reach under the occlusion board “as quickly and accurately as possible” to the remembered location of the target. This memory-guided reaching was implemented to help prevent the early onset of error-related ERPs to premature visual feedback provided by the emergence of the participant’s hand from the occlusion board in comparison with the target (MacLean et al., 2015). At screen touch, participants received visual feedback (differing by experiment, see Figure 1) and were instructed to hold their finger where it landed until they heard another auditory cue (1,000 Hz, 0.05 s, 30 dB) at 1,000 ms after screen-touch. This cue signaled that the trial was complete and that they could return to the spacebar to initiate the next trial^1^. Trials in the PVSA blocks were similar except that participants could always see the target during the entire reach, so the auditory go-cue to reach sounded 700–900 ms after the centrally located target appeared.

### Behavioral Data Collection and Analysis

Pointing error size was recorded as the horizontal distance between the location of the screen touch and the target, measured in pixels and converted to visual degrees. Distances to the left of the target were recorded as negative values, and distances to the right of the target were recorded as positive values. For statistical analyses, blocks were divided into six phases of adaptation (trials 1–10, 11–20, 21–30, 31–40, 41–50, 51–60; P1 to P6), consistent with the bins of phases used to measure ERPs (described below). Absolute errors were then submitted to a 2 × 2 × 6 repeated-measures ANOVA with the following factors: exposure type (prism, sham), shift direction (left, right), and phase (P1 to P6). Note that sham blocks were coded according to the shift direction of the preceding prism block, and the baseline block was omitted from this analysis for not having a shift direction. For Experiments 1 and 3, there were no significant effects of shift direction on absolute error magnitude (*p*s ≥ 0.1), so all results for those experiments were collapsed across this factor. For Experiment 2, the three-way interaction between exposure type, phase, and shift direction was significant (*p* = 0.003). Although subsequent ANOVAs showed a significant phase-by-shift direction interaction for prism exposure but not sham exposure, *post hoc* analyses did not reveal any significant differences in absolute errors. Thus, subsequent analyses were also collapsed across shift direction for Experiment 2. To determine if there were differences across exposure type and phase for each experiment, the absolute errors were submitted to a 3 × 6 repeated-measures ANOVA with the following factors: exposure type (prism, sham, baseline), and phase (P1 to P6). Absolute errors were also compared across experiments in a 3 × 6 × 3 mixed ANOVA of exposure type, phase, and experiment (1, 2, 3).

Aftereffects were measured by subtracting average error size during baseline PVSA from average error size during post-PA PVSA blocks. Absolute values of these baseline-corrected aftereffects were compared across left- and right-shifting prism directions using paired samples *t*-tests. Since aftereffects did not differ by shift direction for any experiment (*p*s ≥ 0.42), these aftereffects were averaged across shift direction and compared across the three experiments using one-way ANOVA.

### Electroencephalography Data Analysis

Data were processed offline using the EEGLAB toolbox (Delorme and Makeig, 2004), and its extension ERPLAB (Lopez-Calderon and Luck, 2014). First, raw EEG data were visually inspected and channels showing abnormal activity (e.g., dead, noisy, frequent large artifacts) were removed. Next, data were filtered (IIR Butterworth) using a high-pass of 0.1 Hz, a low-pass of 30 Hz, and a 24 dB/oct roll-off. All data were then re-referenced to the average of the two mastoid channels, after which the mastoid channels were removed from the data. After segmenting into 1,100 ms epochs (300 ms pre- to 800 ms post-screen touch), any channel that had a mean voltage of five or more standard deviations from the joint probability of all channels was removed. Also, any epoch showing mean voltage of six or more standard deviations from either the within-channel mean, or across-channel mean for that epoch was removed. The remaining data were submitted to an Independent Component Analysis (ICA) using the runica function in EEGLAB. Components reflecting ocular artifacts (e.g., blinks, saccades) were manually rejected. Then, an artifact rejection was performed on the data such that any epoch involving a change in voltage that exceeded 100 μV, or any sample-to-sample voltage change exceeding 10 μV was removed from the data. Finally, removed channels were interpolated and segments were averaged together based on events of interest. The overall percentages of ERP data removed before final analyses for Experiments 1, 2, and 3 were 6%, 4%, and 5%, respectively. For all three experiments, the highest percentage of rejected trials for a given participant was 13%.

Only data from prism blocks were used to calculate ERPs. Accuracy was grouped according to three levels: hit (within the target’s 1.3 cm width), small miss (within 2.6 cm of the target’s edge on either side), or big miss (beyond 2.6 cm of the target’s edge on either side). Grouping accuracy according to three levels (hits, small misses, big misses) enabled investigation of ERPs correlating with error magnitude (Vocat et al., 2011; MacLean et al., 2015). The exposure phase was grouped according to six phases (P1 to P6): trials 1–10, 11–20, 21–30, 31–40, 41–50, and 51–60, allowing for investigation of changes in ERP amplitude throughout the adaptation. For the percentage of “hit,” “small miss,” and “big miss” responses by phase, see Supplementary Tables S1–S3.

ERPs were identified based on inspection of difference waveforms, as done previously (Krigolson et al., 2013, 2014). To investigate error-related components, all hit data were subtracted from all miss data (i.e., both “small misses” and “big misses”). To investigate phase-related components, phase 6 data were subtracted from phase 1 data. These difference waveforms for accuracy or phase were calculated for each electrode site, and 95% CIs were inspected to determine if any time points differed significantly from zero. Visual inspection of these difference waveforms yielded: (1) electrode sites displaying maximal differences between levels of accuracy/phase; and (2) time-windows corresponding to the maximal differences between levels of accuracy/phase. Mean voltages were calculated for each accuracy group (hits, small misses, big misses) or phase (P1–P6), at ±50 ms surrounding the identified peak of the difference waveform (there were some exceptions to the ±50 ms time window, as indicated in the “Results” section). To determine whether a given ERP was evoked, we submitted these means to a one-way repeated-measures ANOVA with accuracy or phase as a factor. The ERP component means were also submitted to a contrast analysis to determine if the mean voltage showed a linear trend for either factor.

### Statistical Analysis

Analyses were conducted using IBM SPSS Statistics (v.25) and GraphPad Prism (v.6). *p* < 0.05 was considered statistically significant, and error bars in figures represent standard error of the mean. When a repeated-measures ANOVA revealed a significant effect, pairwise comparisons between each level were completed using the Bonferroni adjustment. Greenhouse-Geisser corrected degrees of freedom were used in reporting all statistical tests that did not meet the assumption of sphericity (i.e., Mauchly’s test *p* < 0.001).

## Results

### Experiment 1: Target and Direct Hand Feedback

#### Summary of Hypotheses

Experiment 1 was a partial replication of MacLean et al. (2015) in that participants received both target and direct hand feedback at screen touch (see Figure 1). We hypothesized that this feedback would evoke both an FRN sensitive to pointing error size, and a P300 decreasing in amplitude throughout the adaptation.

#### Exclusions

From an initial sample of 20, two participants were excluded from data analysis because of poor EEG data quality resulting in high artifact rejection (>50%), leaving *N* = 18 for the analyses below.

#### Behavioral Results

**Figure 2:**
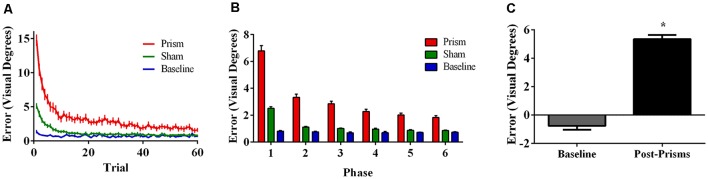
Behavioral results from Experiment 1 (target and direct hand feedback; *N* = 18). The figures show **(A)** average pointing error size across the 60 trials for prism, sham, and baseline blocks (left); **(B)** the same variables represented as model-predicted estimated marginal means (middle); and **(C)** pointing error size on proprioceptive-visual straight ahead (PVSA) blocks at baseline and after PA (right; *p < 0.001). Error bars represent standard error of the mean.

Error size showed main effects of phase (*F*_(5, 85)_ = 166.73, *p* < 0.001, ε = 0.38, partial *η*^2^ = 0.91) and exposure type (*F*_(2, 34)_ = 214.67, *p* < 0.001, ε = 0.56, partial *η^2^* = 0.93), and an interaction between phase and exposure type (*F*_(10,170)_ = 76.77, *p* < 0.001, ε = 0.26, partial *η^2^* = 0.82). *Post hoc* analyses revealed that error size for the baseline block did not differ across phase. For prism blocks, error size decreased from phase 1 through 3 and leveled off at phase 4, indicating adaptation to the visual shift. For sham blocks, error size decreased from phase 1–2 and reached baseline levels by phase 4, indicating successful de-adaptation (Figure 2A,B). Finally, the prism blocks produced significant aftereffects (*t*_(17)_ = 13.50, *p* < 0.001) with an average (baseline-corrected) magnitude of 5.34 visual degrees (*SEM* = 0.29; Figure 2C).

#### Electroencephalography Results

**Figure 3:**
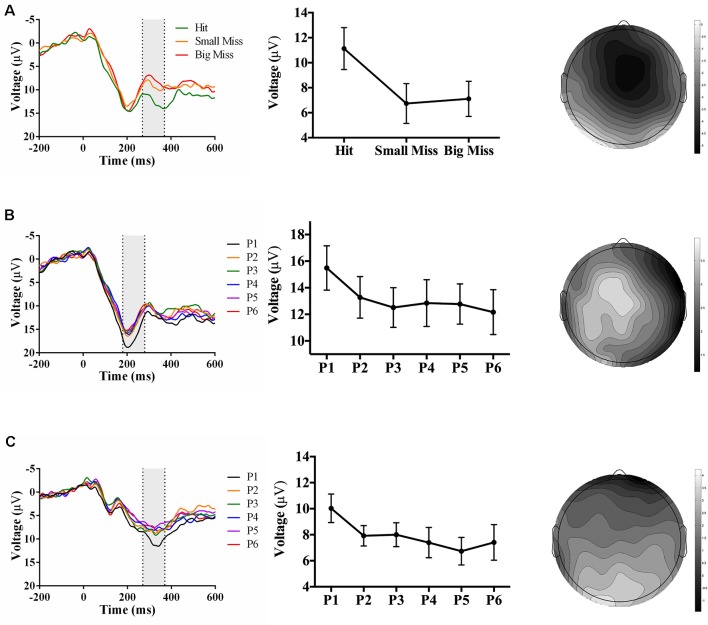
Waveforms (left column), contrast analyses (middle column), and scalp topographies (right column) of event-related potential (ERP) findings from Experiment 1 (target and direct hand feedback; *N* = 18). Analyses revealed: (**A**; top row) a negative-going component sensitive to accuracy (*p* < 0.001), appearing at FCz and at 270–370 ms post-screen touch; (**B**; middle row) a positive-going component at sensitive to phase (*p* = 0.005), appearing at Cz and at 180–280 ms post-screen touch; and (**C**; bottom row) another positive-going component sensitive to phase (*p* = 0.05), appearing at Oz and at 270–370 ms post-screen touch. Scalp topography scale bars are in μV; error bars represent standard error of the mean.

As predicted, analyses revealed an accuracy-sensitive component maximal at electrode FCz and 270–370 ms post-screen touch, *F*_(2, 34)_ = 16.56, *p* < 0.001, partial *η^2^* = 0.49. *Post hoc* analyses showed that both “small misses” and “big misses” had more negative amplitudes than “hits” (*p* < 0.05). Amplitudes also showed a linear trend over accuracy level (*F*_(1,17)_ = 14.72, *p* = 0.001, partial *η^2^* = 0.46), likely driven by the decrease in voltage from “hit” to “small miss” (Figure 3A). The electrode site, latency, and evoking stimulus (i.e., visual feedback on miss trials) all suggest that this component represents an FRN sensitive to pointing accuracy during PA (Miltner et al., 1997; Holroyd and Coles, 2002; Krigolson and Holroyd, 2007).

Analyses also revealed two phase-sensitive components: at electrode Cz, the mean peak voltage 180–280 ms post-screen touch was sensitive to phase, *F*_(5, 85)_ = 3.67, *p* = 0.005, partial *η^2^* = 0.18. *Post hoc* analyses showed that phase 1 had a more positive amplitude than phases 3 and 4 (*p* < 0.05). Contrast analyses suggested that amplitudes decreased linearly from phase 1–6 (*F*_(1, 17)_ = 14.72, *p* = 0.001, partial *η^2^* = 0.46; Figure 3B). Although the latency of this component was too early to be a P300, it could instead reflect a P2 component, which is linked to early attentional processing (Luck, 2005). Finally a component maximal at electrode site Oz and occurring 270–370 ms post-screen touch was also sensitive to phase, *F*_(5, 85)_ = 2.90, *p* = 0.05, partial *η^2^* = 0.15. *Post hoc* analyses did not reveal any significant differences, but the linear trend of decreasing amplitude from phase 1–6 approached significance, *F*_(1, 17)_ = 4.10, *p* = 0.06, partial *η^2^* = 0.19 (Figure 3C). The latency of this posterior positivity was earlier and more posterior than what is typically reported for the P300 (Polich and Kok, 1995; MacLean et al., 2015).

### Experiment 2: Direct Hand Feedback Only

#### Summary of Hypotheses

The target and direct hand visual feedback in Experiment 1 evoked components resembling the FRN and the P300, and also produced robust aftereffects. To help disentangle which aspect of the visual feedback (i.e., the re-appearance of the target, or the direct view of the hand) was more strongly linked to the ERPs and aftereffects, Experiments 2 and 3 employed each type of feedback separately. In Experiment 2, the participants saw their hand at screen-touch, but no re-appearance of the target line (i.e., direct hand feedback only). Because participants could not directly see their pointing accuracy in relation to the target’s position, it was hypothesized that an FRN would not be evoked. By contrast, given the apparent role of viewing the hand in producing successful adaptation (Clower and Boussaoud, 2000; Wilms and Malá, 2010; Veilleux and Proteau, 2015), we hypothesized that the condition would still produce strong aftereffects, in addition to a phase-sensitive P300 thought to be related to spatial realignment (MacLean et al., 2015).

#### Exclusions

One participant’s behavioral data was unusable due to a recording error. This participant’s behavior during the experiment was observed to be consistent with other participants, thus her EEG data were kept in the analysis to increase power. As a result, the behavioral analysis reflects 21 participants, whereas the EEG analysis reflects 22 participants.

#### Behavioral Results

**Figure 4:**
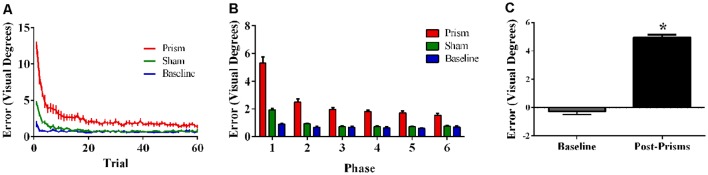
Behavioral results from Experiment 2 (direct hand feedback only; *N* = 21). The figures show **(A)** average pointing error size across the 60 trials for prism, sham, and baseline blocks (left); **(B)** the same variables represented as model-predicted estimated marginal means (middle); and **(C)** pointing error size on proprioceptive-visual straight ahead (PVSA) blocks at baseline and after PA (right; *p < 0.001). Error bars represent standard error of the mean.

Error size showed main effects of phase (*F*_(5,100)_ = 85.62, *p* < 0.001, ε = 0.30, partial *η^2^* = 0.81) and exposure type (*F*_(2, 40)_ = 133.37, *p* < 0.001, ε = 0.55, partial *η^2^* = 0.87), and an interaction between phase and exposure type (*F*_(10, 200)_ = 36.96, *p* < 0.001, ε = 0.17, partial *η^2^* = 0.65). *Post hoc* analyses revealed that error size for the baseline block did not differ across phase. For prism blocks, error size decreased from phase 1–2 and leveled off at phase 3, indicating adaptation to the visual shift. For sham blocks, error size decreased from phase 1–2 and reached baseline levels by phase 4, indicating successful de-adaptation (Figures 4A,B). Finally, the prism blocks produced significant aftereffects (*t*_(20)_ = 18.31, *p* < 0.001), with an average (baseline-corrected) magnitude of 4.95 visual degrees (*SEM* = 0.19; Figure 4C).

#### Electroencephalography Results

**Figure 5:**
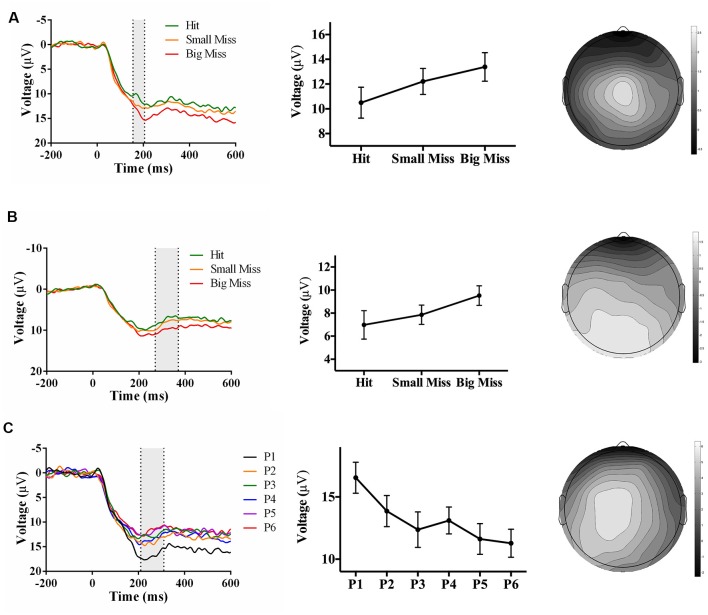
Waveforms (left column), contrast analyses (middle column), and scalp topographies (right column) of ERP findings from Experiment 2 (direct hand feedback only; *N* = 22). Analyses revealed three positive-going components: (**A**; top row) one component sensitive to accuracy (*p* = 0.001), appearing at Cz and at 155–205 ms post-screen touch; (**B**; middle row) one component sensitive to accuracy (*p* = 0.001), appearing at POz and at 270–370 ms post-screen touch; and (**C**; bottom row) one component sensitive to phase (*p* < 0.001), appearing at CPz and 210–310 ms post-screen touch. Scalp topography scale bars are in μV; error bars represent standard error of the mean.

In contrast to Experiment 1, there was no negative-going accuracy-sensitive component in Experiment 2. Instead, two positive-going components were found. The first accuracy-sensitive component was maximal at electrode Cz and 155–205 ms post-screen touch, *F*_(2, 42)_ = 8.36, *p* = 0.001, partial *η^2^* = 0.28. *Post hoc* analyses showed that “big misses” had more positive amplitudes than “hits” (*p* < 0.05). Amplitudes also showed a linear trend over accuracy level (*F*_(1, 17)_ = 14.72, *p* = 0.001, partial *η^2^* = 0.46), suggesting that the positive amplitude increased concomitantly with error size (Figure 5A). The electrode site, latency, and evoking stimulus (i.e., visual feedback on miss trials) suggest that this positive component may reflect an error positivity (Pe), which was sensitive to accuracy during PA in prior work (Vocat et al., 2011). The second accuracy-sensitive component was maximal at electrode POz and 270–370 ms post-screen touch, *F*_(2, 42)_ = 8.14, *p* = 0.001, partial *η^2^* = 0.28. *Post hoc* analyses showed that big misses” had more positive amplitudes than “hits” or “small misses” (*p* < 0.05), and the contrast analysis suggested that voltage increased with error size, *F*_(1, 21)_ = 13.61, *p* = 0.001, partial *η^2^* = 0.39 (Figure 5B). This component could represent a late Pe, or a P300, although the latency was earlier and electrode site more posterior than typically reported (Krigolson and Holroyd, 2007; Krigolson et al., 2008).

Analyses also revealed a positive-going phase-sensitive component maximal at electrode CPz and 210–310 ms post-screen touch, *F*_(5,105)_ = 6.92, *p* < 0.001, partial *η^2^* = 0.25. *Post hoc* analyses showed that phase 1 had a more positive amplitude than phases 3, 4, and 6 (*p* < 0.06), and contrast analyses suggested that amplitudes decreased linearly from phase 1–6, *F*_(1,21)_ = 14.23, *p* = 0.001, partial *η^2^* = 0.40 (Figure 5C). The latency and electrode site of this component is broadly consistent with a P300 sensitive to the phase of adaptation, albeit significantly earlier than typically reported (Polich and Kok, 1995; MacLean et al., 2015).

### Experiment 3: Target and Indirect Hand Position Feedback

#### Summary of Hypotheses

In Experiment 3, participants could not directly view their hand (i.e., they reached under full occlusion) and instead saw re-appearance of the black target line and a second light gray vertical line that referred to the hand position below the board. We hypothesized that this feedback would evoke an accuracy-sensitive FRN due to the provision of explicit feedback about pointing accuracy in relation to the target position. By contrast, based on prior studies suggesting that direct view of the hand is important for successful sensorimotor realignment (Clower and Boussaoud, 2000; Wilms and Malá, 2010; Veilleux and Proteau, 2015), we expected that this condition would produce weaker aftereffects than the other two experiments, and the phase-sensitive P300, if it reflects a realignment process, would be absent.

#### Exclusions

Three participants were excluded due to poor EEG data quality resulting in high artifact rejection (>50%). Additionally, one participant’s behavioral data were lost due to a recording error, although her EEG data were still available. This participant’s behavior during the experiment was observed to be consistent with other participants, thus her EEG data were kept in the analysis to increase power. As a result, the behavioral analysis reflects 21 participants, whereas the EEG analysis reflects 22 participants.

#### Behavioral Results

**Figure 6:**
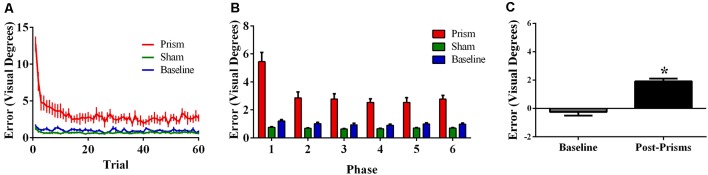
Behavioral results from Experiment 3 (target and indirect hand position feedback; *N* = 21). The figures show **(A)** average pointing error size across the 60 trials for prism, sham, and baseline blocks (left); **(B)** the same variables represented as model-predicted estimated marginal means (middle); and **(C)** pointing error size on proprioceptive-visual straight ahead (PVSA) blocks at baseline and after PA (right; *p < 0.001). Error bars represent standard error of the mean.

Error size showed main effects of phase (*F*_(5, 100)_ = 27.76, *p* < 0.001, ε = 0.42, partial *η^2^* = 0.58) and exposure type (*F*_(2, 40)_ = 45.00, *p* < 0.001, ε =.52, partial *η^2^* = 0.69), and an interaction between phase and exposure type (*F*_(10, 200)_ = 20.80, *p* < 0.001, ε = 0.21, partial *η^2^* = 0.51). *Post hoc* analyses revealed that error size for the baseline block did not differ across phase. For prism blocks, error size decreased from phase 1–2 and leveled off, indicating adaptation to the visual shift. For sham blocks, error size decreased from phase 1–2 and leveled off, indicating some de-adaptation. Error size of sham blocks was significantly lower than baseline across all six phases, suggesting some practice effects (Figures 6A,B). Finally, the prism blocks produced significant aftereffects (*t*_(21)_ = 6.79, *p* < 0.001), with an average (baseline-corrected) magnitude of 1.92 visual degrees (*SEM* = 0.19; Figure 6C).

#### Electroencephalography Results

**Figure 7:**
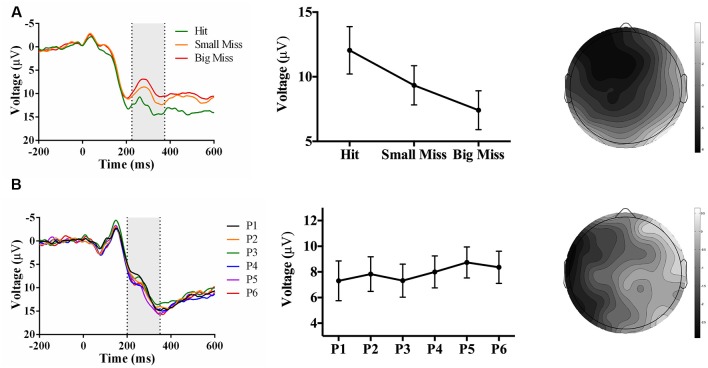
Waveforms (left column), contrast analyses (middle column), and scalp topographies (right column) of ERP findings from Experiment 3 (target and indirect hand position feedback; *N* = 22). Analyses revealed (**A**; top row) a negative-going component sensitive to accuracy (*p* < 0.001), appearing at FCz and at 225–375 ms post-screen touch. No phase-sensitive components were found by visual inspection of difference waveforms, and also illustrated by (**B**; bottom row) the lack of differences by phase at electrode site POz and 200–350 ms post-screen touch. Scalp topography scale bars are in μV; error bars represent standard error of the mean.

As predicted, analyses revealed an accuracy-sensitive component maximal at electrode FCz and 225–375 ms post-screen touch, *F*_(2, 42)_ = 27.85, *p* < 0.001, partial *η^2^* = 0.57. *Post hoc* analyses showed that all three accuracy levels differed from each other (*p* < 0.05); namely, contrast analyses suggested that amplitudes became increasingly negative as error size increased, *F*_(1, 21)_ = 40.40, *p* = 0.017, partial *η^2^* = 0.66 (Figure 7A). The electrode site, latency, and evoking stimulus (i.e., visual feedback on miss trials) all suggest that this component represents an FRN sensitive to accuracy during PA (Miltner et al., 1997; Holroyd and Coles, 2002).

No phase-sensitive components were identified. To confirm this null result, we conducted a repeated-measures ANOVA of phase (P1 to P6) and electrode site (Fpz, FCz, Cz, CPz, Pz, POz, Oz) predicting mean peak voltage 200–350 ms post-screen touch, which found no effect of phase or interaction between phase and electrode site (*p*s ≥ 0.28; Figure 7B).

#### Comparison of Behavior Across Experiments

With error size during the exposure phase as the outcome, analyses revealed a three-way interaction between exposure type, phase, and experiment, *F*_(20, 570),_ ε = 0.22 = 4.12, *p* = 0.003, partial *η^2^* = 0.13 (all main effects and two-way interactions were also significant, *p*s ≤ 0.014). To explore the nature of the interaction, a series of two-way ANOVAs of phase and experiment were conducted. In the baseline block, there was a main effect of experiment (*F*_(2, 57)_ = 10.52, *p* < 0.001, partial *η^2^* = 0.27), whereby errors were significantly larger in Experiment 3 than in Experiments 1 or 2 (*p*s ≤ 0.002). In the prism blocks, there was an interaction between phase and experiment (*F*_(10,285),_ ε = 0.36 = 4.01, *p* = 0.006, partial *η^2^* = 0.12), whereby errors were significantly larger in Experiment 3 than in Experiments 1 or 2, but only in phase 6 (*p*s ≤ 0.004). In the sham blocks, there was also an interaction between phase and experiment (*F*_(10, 285),_ ε = 0.46 = 57.88, *p* < 0.001, partial *η^2^* = 0.67): for phases 1 through 4, errors were generally largest in Experiment 1, followed by Experiment 2 and then Experiment 3; however, for phases 5 and 6, errors did not significantly differ across experiments (*p*s ≥ 0.14).

**Figure 8:**
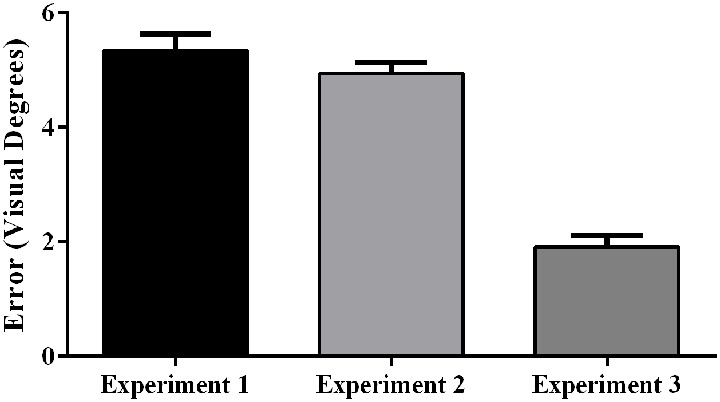
Aftereffects by experiment, showing weaker aftereffects in Experiment 3 (target and indirect hand position feedback) than in Experiment 1 (target and direct hand feedback) or Experiment 2 (direct hand feedback only;* p* < 0.001). Error bars represent standard error of the mean.

Aftereffect size also differed significantly across experiments, *F*_(2, 57)_ = 72.25, *p* < 0.001, partial *η^2^* = 0.72. *Post hoc* analyses revealed that the aftereffects in Experiment 3 were smaller than the aftereffects in Experiments 1 and 2 (Figure 8).

## Discussion

The objective of the present study was to investigate candidate neural events contributing to strategic recalibration (the FRN) and spatial realignment (the P300) by employing different types of visual feedback during PA exposure. We hypothesized that: visual feedback involving direct view of the hand together with a re-appearance of the target position would evoke a phase-sensitive P300, an accuracy-sensitive FRN, and strong aftereffects (Experiment 1); visual feedback involving direct view of the hand only with no explicit feedback comparing the hand position to target position would not evoke an FRN, but would still evoke the P300 and produce strong aftereffects (Experiment 2), and visual feedback involving view of the target position and indirect hand position would evoke an FRN, but not a P300 and would produce weaker aftereffects than the other two experiments (Experiment 3). Overall, our results generally supported these hypotheses with some differences, all of which are discussed in further detail below.

## Prism Aftereffects in Relation to Visual Feedback

As predicted, participants experienced significantly larger aftereffects when they received visual feedback involving a direct view of the hand (Experiments 1 and 2) than when they received only indirect feedback regarding hand position (Experiment 3). This finding is consistent with prior work documenting larger aftereffects when participants viewed their own hand during PA exposure than when they viewed a “symbolic” hand (e.g., cursor icon; Clower and Boussaoud, 2000; Wilms and Malá, 2010; Veilleux and Proteau, 2015). Figure 6 illustrates that the smaller aftereffects noted in Experiment 3 were likely due to insufficient adaptation during prism exposure. Specifically, pointing errors were significantly larger at the end of the prism blocks in Experiment 3 when compared to Experiments 1 and 2, suggestive of reduced adaptation to the prismatic shift in Experiment 3. Also, Experiments 1 and 2 showed an initial elevation in error size at the beginning of sham blocks (i.e., residual aftereffect) that subsequently dropped to baseline levels as participants de-adapted. By contrast, sham blocks in Experiment 3 started at baseline error levels, which also indicates weaker adaptation to the prior visual shift.

If we consider the magnitude of aftereffects as an index of spatial realignment, these results are consistent with the interpretation that direct view of the hand during PA exposure facilitates realignment processes (Redding et al., 2005; Redding and Wallace, 2006). Furthermore, the magnitude of aftereffects was not different between Experiments 1 and 2, which differed only in whether the target line re-appeared at screen touch. This finding suggests that receiving explicit error feedback during prism exposure that compares the hand’s position to the target’s position is not critical to producing strong aftereffects.

## Electroencephalography: Accuracy-Sensitive Components

As hypothesized, the event of screen touch with a direct comparison of the hand’s position to the target’s prior position (Experiments 1 and 3) evoked a negative-going component at electrode site FCz and ~225–375 ms post-screen touch that was sensitive to pointing accuracy. This electrode site, timing, and evoking stimulus (i.e., the onset of error feedback) are consistent with the properties of an FRN (Miltner et al., 1997; Holroyd and Coles, 2002; Krigolson and Holroyd, 2007). Given that both Experiments 1 and 3 evoked an FRN, but only Experiment 1 yielded strong aftereffects, the neural process underlying the FRN may not be essential to producing strong aftereffects. Moreover, screen touch with no re-appearance of the target line (Experiment 2) did not evoke an FRN component, but still produced strong aftereffects. This result suggests that when there was no stimulus available to explicitly indicate the magnitude of reaching errors (i.e., by displaying the discrepancy between hand location and target location at screen touch), participants did not undergo the type of error processing that generates an FRN component; however, they still engaged in effective realignment, as evidenced by their large aftereffects (Redding et al., 2005). Overall, these findings support the theory that the FRN may contribute to the “strategic recalibration” process that is involved in error processing during prism exposure but is not as central to the production of PA aftereffects (Redding and Wallace, 1993; Michel et al., 2007; MacLean et al., 2015). Finally, it is important to note that the correlation between FRN amplitude and error size does not indicate that the FRN is directly causing recalibration. Alternatively, the FRN may reflect the computation of the reaching error (Holroyd and Coles, 2002; Ichikawa et al., 2010). This error information may subsequently inform the recalibration process (i.e., the previously computed error contributes to planning the next reach).

Although providing direct hand feedback only (Experiment 2) did not evoke an FRN, it did evoke positive-going component at electrode site Cz and ~155–205 ms post-screen touch that increased in amplitude with pointing error size. This component may represent a Pe, which typically peaks at 100–200 ms post-response onset over central scalp electrodes and is thought to represent subjective error evaluation (Falkenstein et al., 1991; Wessel, 2012). Notably, our component resembles the Pe identified by Vocat et al. (2011) which was also sensitive to pointing error size during PA. Given the strong aftereffects measured in Experiment 2, we cannot rule out a potential contribution of this Pe to spatial realignment processes.

## Electroencephalography: Phase-Sensitive Components

As hypothesized, visual feedback conditions that provided a direct view of the hand at screen touch (Experiments 1 and 2) evoked positive-going components that decreased in amplitude as adaptation proceeded. While visual feedback in Experiment 2 evoked a phase-sensitive component with latency and topography that were broadly consistent with the P300 identified by MacLean et al. (2015), visual feedback in Experiment 1 elicited two positive-going phase-sensitive components: an earlier component (~180–280 ms post-screen touch) maximal at electrode Cz, and a later component (~270–370 ms post-screen touch) maximal at electrode Oz. The timing and relative topography of these components resemble Polich’s (2007) proposed P3a and P3b subcomponents of the P300. If that is the case, then the first component may reflect initial attentional processing of the visual feedback, and the second component may reflect the integration of that information into the participant’s inner working model of the environment, in line with the context-updating hypothesis (Donchin and Coles, 1988, 1998; Polich, 2007). Overall, results from Experiments 1 and 2 suggest that direct view of the hand at reach termination (regardless of whether feedback regarding prior target location is also provided) is associated with a P300-like component that decreases in amplitude across phase of adaptation. Importantly, the direct view of the hand also produced the largest aftereffects, whereas indirect feedback regarding hand position (Experiment 3) produced significantly weaker aftereffects, and an absence of phase-sensitive positive-going components. Thus, the P300 component may reflect neural processing of a mismatch between visually perceived hand location and proprioceptively felt hand location; resolving this discrepancy through successful realignment may in turn result in a smaller P300 over time. As such, the P300 may index a “spatial realignment” process that is thought to give rise to larger aftereffects following glasses removal (Redding et al., 2005; Redding and Wallace, 2006).

## Connection to Neuroanatomical Theories of Adaptation

Taken together, our results provide evidence for the proposal that the processes underlying the FRN and P300 contribute to the PA processes of strategic recalibration and spatial realignment, respectively (Redding and Wallace, 1996; MacLean et al., 2015). In further support of these ERP correlates, the hypothesized neural generators of the FRN and P300 overlap with brain regions that have been putatively associated with recalibration and realignment. For instance, numerous studies have source-localized the FRN to the anterior cingulate cortex (ACC; Miltner et al., 1997; Holroyd et al., 2004). This brain region also displays increased activity during the early phases of PA exposure while participants are making errors (Danckert et al., 2008). Another study identified that frontal midline theta dynamics time-locked to visual feedback during PA were modulated by error magnitude (Arrighi et al., 2016). Although the proposed neural generators of the P300 are varied (Polich and Kok, 1995; Polich, 2007), its more posterior topography is broadly consistent with the proposed role of the posterior parietal cortex in realignment processes (Chapman et al., 2010). Also, a recent study involving sensorimotor adaptation to a rotation task demonstrated that the amplitude of the P300 at the onset of sensory feedback correlated with participants’ learning rate (Palidis et al., 2019). Overall, the overlap between the brain regions associated with the FRN and P300 and recent neuroanatomical accounts of visuomotor adaptation reinforces the theory that these components may index neural processes that are relevant to PA.

More broadly, there is growing evidence to suggest that successful visuomotor learning relies on a distributed frontoparietal network, whereby frontal regions coordinate online movement corrections, and parietal regions contribute to a more gradual adaptation that involves learning from past corrections (Mutha et al., 2011a,b, 2014; Arrighi et al., 2016). These two processes resemble the strategic recalibration and spatial realignment processes of PA (Redding and Wallace, 1996), and their neural correlates resemble the regions underlying our ERPs of interest. Recent neural theories specific to PA include an even broader network of brain regions, including the cerebellum, motor cortices, and temporal areas (Panico et al., 2020). The cerebellum, in particular, has been implicated as a neural substrate of recalibration and realignment across studies (Chapman et al., 2010; Küper et al., 2014), and cerebellar lesions have been associated with incomplete or absent adaptation (Martin et al., 1996; Baizer et al., 1999; Morton and Bastian, 2004). Based on our study, we cannot specify the cerebellum’s involvement in interpreting our visual feedback conditions; more generally, our ERP methodology does not allow us to make firm conclusions about localization of neural functions underlying PA. In the future, studies recording ERPs and functional activity simultaneously during PA (e.g., MRI-compatible EEG) could more fully investigate how PA modulates these complex neural functions and networks.

## Limitations

While novel in identifying promising neural correlates of PA, our study is not without limitations. First, the present study focused on the FRN and P300 as markers of dissociable PA processes (i.e., recalibration and realignment; Redding and Wallace, 1996). However, given their similar timing and shared modulation by stimulus frequency, these two components can be subject to cross-contamination. For instance, the FRN’s amplitude can be diminished by a subsequent P300 response when an infrequent stimulus is presented (Krigolson, 2018). Because errors become less frequent between early and late phases of prism exposure, it is challenging to distinguish neural markers of errors and phase during PA. Indeed, the overlap between the FRN and P300 could explain why a *positive*-going accuracy-sensitive component with timing resembling the P300 was measured in Experiment 2 (that said, if the P300 was primarily a response to errors that were interpreted as infrequent stimuli, its amplitude would not be expected to diminish across the exposure phase as errors became less frequent, which is what was observed in Experiments 1 and 2).

Second, we used terminal exposure during PA to minimize the potential for premature neural responses to visual feedback from viewing the hand during the reaching trajectory (MacLean et al., 2015). Despite this procedure, terminal exposure still provided participants with some premature visual information regarding their hand’s position in the last 3 cm before screen touch. This early visual feedback could explain why the phase-sensitive components measured in Experiments 1 and 2 had earlier latencies than what is typically reported for a P300 (Polich and Kok, 1995; Luck, 2005; MacLean et al., 2015). One approach to overcoming this limitation could be to occlude participants’ vision until the moment they contact the screen using special goggles (e.g., PLATO goggles; see Striemer and Borza, 2017). It is unclear whether eliminating all visual input during the reach portion of each trial would impact PA’s effects more broadly, but future studies could investigate this possibility. An alternative approach would be to use motion-capture technology to determine the precise moment when the participant’s hand becomes visible to them (O’Shea et al., 2014; MacLean et al., 2015).

## Conclusions and Future Directions

The present study sought to identify ERP correlates of strategic recalibration and spatial realignment during PA. Participants reached for vertical line targets on a touch screen in alternating prism and sham blocks under terminal exposure, and we manipulated visual feedback provided at screen touch. Results showed that a direct view of the reaching hand at screen touch was both critical to obtaining large aftereffects and associated with a P300-like component that decreased in amplitude throughout the adaptation. This ERP component may reflect a context-updating process in the parietal cortex that is important for the realignment of sensorimotor reference frames and strong aftereffects (Donchin and Coles, 1988, 1998; Chapman et al., 2010). By contrast, visual feedback showing an explicit mismatch between the hand and target position was associated with an FRN, but this component alone was not sufficient to obtain strong aftereffects. Thus, the FRN may reflect a mid-frontal learning system primarily important for goal-directed error compensation and motor learning (i.e., recalibration; Krigolson et al., 2008; Anguera et al., 2009), but not sufficient for optimal sensorimotor realignment.

These findings not only advance our understanding of the neural processes engaged by PA, but they can also inform future studies of neural responses to PA in healthy older adults and stroke patients with visuospatial neglect. While PA has been deemed one of the most promising treatments for neglect, the neural mechanisms of PA-induced neglect recovery require further investigation (Yang et al., 2013; Rossetti et al., 2019). For instance, no studies to date have examined ERPs in stroke patients with neglect undergoing PA. However, functional magnetic resonance imaging (fMRI) data show that brain areas that are implicated in the FRN and P300 (e.g., frontal and parietal regions) are also activated by PA in neglect patients (Saj et al., 2013). Also, patients with neglect display reduced P300 amplitudes and increased latencies during visuospatial orienting paradigms (Saevarsson et al., 2012; Ye et al., 2019). These findings, combined with results from the present study, provide the groundwork for investigating the FRN and the P300 concerning PA response in stroke patients with neglect.

## Data Availability Statement

The raw data supporting the conclusions of this article will be made available by the authors, without undue reservation, to any qualified researcher.

## Ethics Statement

The studies involving human participants were reviewed and approved by Nova Scotia Health Authority Research Ethics Board. The patients/participants provided their written informed consent to participate in this study.

## Author Contributions

SM, OK, and GE contributed to designing the study and collecting the data. JA and SM analyzed the data. All authors interpreted the results. JA and SM wrote a draft of the manuscript. All authors reviewed and revised the manuscript and confirmed its final version.

## Conflict of Interest

The authors declare that the research was conducted in the absence of any commercial or financial relationships that could be construed as a potential conflict of interest.

## Footnotes

^1^Trials in the sham blocks had the same event sequence as the prism blocks, except that immediate target feedback (i.e., at screen touch) and delayed target feedback (i.e., 750-850 ms after screen touch) were randomized across each trial. This varied feedback was used to facilitate comparison with a separate experiment not reported in this article. These sham blocks were successful in producing de-adaptation (see “Results” section).
