# Supplementary material for: Visual Feedback Modulates Aftereffects and Electrophysiological Markers of Prism Adaptation
Source: Front Hum Neurosci. 2020 Apr 17;14:138. doi: 10.3389/fnhum.2020.00138 (PMC7182100; doi:10.3389/fnhum.2020.00138)
Supplement: Supplementary file 1 [file Table_1.DOCX]

Supplementary Material

# Supplementary Tables

Table S1

*Percentage of Accuracy Response Types by Phase and Exposure for Experiment 1*

| Exposure | Phase | Hits (%) | Small misses (%) | Large misses (%) |
| --- | --- | --- | --- | --- |
| Baseline | 1 | 53 | 44 | 2 |
|  | 2 | 56 | 43 | 2 |
|  | 3 | 67 | 30 | 3 |
|  | 4 | 64 | 34 | 2 |
|  | 5 | 62 | 37 | 1 |
|  | 6 | 61 | 39 | 1 |
| Prism | 1 | 6 | 13 | 80 |
|  | 2 | 10 | 27 | 63 |
|  | 3 | 13 | 30 | 57 |
|  | 4 | 19 | 39 | 42 |
|  | 5 | 23 | 41 | 36 |
|  | 6 | 27 | 42 | 31 |
| Sham | 1 | 17 | 34 | 50 |
|  | 2 | 40 | 49 | 10 |
|  | 3 | 45 | 48 | 7 |
|  | 4 | 48 | 45 | 7 |
|  | 5 | 54 | 41 | 5 |
|  | 6 | 53 | 43 | 4 |

Table S2

*Percentage of Accuracy Response Types by Phase and Exposure for Experiment 2*

| Exposure | Phase | Hits (%) | Small misses (%) | Large misses (%) |
| --- | --- | --- | --- | --- |
| Baseline | 1 | 56 | 39 | 6 |
|  | 2 | 64 | 32 | 3 |
|  | 3 | 64 | 35 | 1 |
|  | 4 | 66 | 32 | 2 |
|  | 5 | 66 | 33 | 1 |
|  | 6 | 66 | 31 | 2 |
| Prism | 1 | 10 | 20 | 71 |
|  | 2 | 18 | 35 | 47 |
|  | 3 | 22 | 45 | 34 |
|  | 4 | 24 | 42 | 33 |
|  | 5 | 28 | 39 | 33 |
|  | 6 | 30 | 46 | 24 |
| Sham | 1 | 24 | 42 | 34 |
|  | 2 | 47 | 48 | 5 |
|  | 3 | 59 | 38 | 3 |
|  | 4 | 63 | 34 | 3 |
|  | 5 | 62 | 36 | 2 |
|  | 6 | 58 | 39 | 3 |

Table S3

*Percentage of Accuracy Response Types by Phase and Exposure for Experiment 3*

| Exposure | Phase | Hits (%) | Small misses (%) | Large misses (%) |
| --- | --- | --- | --- | --- |
| Baseline | 1 | 35 | 50 | 15 |
|  | 2 | 47 | 46 | 7 |
|  | 3 | 51 | 42 | 7 |
|  | 4 | 51 | 45 | 4 |
|  | 5 | 45 | 51 | 4 |
|  | 6 | 48 | 46 | 6 |
| Prism | 1 | 15 | 23 | 61 |
|  | 2 | 21 | 33 | 46 |
|  | 3 | 24 | 32 | 44 |
|  | 4 | 25 | 35 | 40 |
|  | 5 | 20 | 42 | 38 |
|  | 6 | 23 | 29 | 49 |
| Sham | 1 | 65 | 30 | 5 |
|  | 2 | 66 | 32 | 2 |
|  | 3 | 69 | 28 | 2 |
|  | 4 | 68 | 31 | 2 |
|  | 5 | 67 | 30 | 3 |
|  | 6 | 66 | 31 | 4 |
